# Age- And Sex-Related Variations in Platelet Count in Italy: A Proposal of Reference Ranges Based on 40987 Subjects' Data

**DOI:** 10.1371/journal.pone.0054289

**Published:** 2013-01-31

**Authors:** Ginevra Biino, Iolanda Santimone, Cosetta Minelli, Rossella Sorice, Bruno Frongia, Michela Traglia, Sheila Ulivi, Augusto Di Castelnuovo, Martin Gögele, Teresa Nutile, Marcella Francavilla, Cinzia Sala, Nicola Pirastu, Chiara Cerletti, Licia Iacoviello, Paolo Gasparini, Daniela Toniolo, Marina Ciullo, Peter Pramstaller, Mario Pirastu, Giovanni de Gaetano, Carlo L. Balduini

**Affiliations:** 1 Institute of Molecular Genetics, National Research Council of Italy, Pavia, Italy; 2 Institute of Population Genetics, National Research Council of Italy, Sassari, Italy; 3 Laboratory of Genetic and Environmental Epidemiology, Research Laboratories, Fondazione di Ricerca e Cura “Giovanni Paolo II”, Università Cattolica. Campobasso, Italy; 4 Institute of Genetic Medicine, EURAC Research, Bolzano, Italy; 5 Institute of Genetics and Biophysics A. Buzzati-Traverso, National Research Council of Italy, Naples, Italy; 6 Division of Genetics and Cell Biology, San Raffaele Scientific Institute, Milano, Italy; 7 Institute for Maternal and Child Health – IRCCS “Burlo Garofolo” – Trieste, Italy; 8 Medical Genetics, Department of Reproductive Sciences, University of Trieste, Trieste, Italy; 9 Laboratory of Cell Biology and Pharmacology of Thrombosis, Reserach Laboratories, Fondazione di Ricerca e Cura “Giovanni Paolo II”, Università Cattolica, Campobasso, Italy; 10 Shardna Life Sciences, Cagliari, Italy; 11 Reserach Laboratories, Fondazione di Ricerca e Cura “Giovanni Paolo II”, Università Cattolica, Campobasso, Italy; 12 Department of Internal Medicine, IRCCS Foundation Policlinico S. Matteo, University of Pavia, Pavia, Italy; University of Leuven, Belgium

## Abstract

**Background and Objectives:**

Although several studies demonstrated that platelet count is higher in women, decreases with age, and is influenced by genetic background, most clinical laboratories still use the reference interval 150–400×10^9^ platelets/L for all subjects. The present study was to identify age- and sex-specific reference intervals for platelet count.

**Methods:**

We analysed electronic records of subjects enrolled in three population-based studies that investigated inhabitants of seven Italian areas including six geographic isolates. After exclusion of patients with malignancies, liver diseases, or inherited thrombocytopenias, which could affect platelet count, reference intervals were estimated from 40,987 subjects with the non parametric method computing the 2.5° and 97.5° percentiles.

**Results:**

Platelet count was similar in men and women until the age of 14, but subsequently women had steadily more platelets than men. The number of platelets decreases quickly in childhood, stabilizes in adulthood, and further decreases in oldness. The final result of this phenomenon is that platelet count in old age was reduced by 35% in men and by 25% in women compared with early infancy. Based on these findings, we estimated reference intervals for platelet count ×10^9^/L in children (176–452), adult men (141–362), adult women (156–405), old men (122–350) and, old women (140–379). Moreover, we calculated an “extended” reference interval that takes into account the differences in platelet count observed in different geographic areas.

**Conclusions:**

The age-, sex-, and origin-related variability of platelet count is very wide, and the patient-adapted reference intervals we propose change the thresholds for diagnosing both thrombocytopenia and thrombocytosis in Italy.

## Introduction

Most laboratories in western countries use the range 150 to 400 or 450×10^9^/L as the reference interval of normal platelet count. These values have been proposed many years ago [{Formatting Citation}1,2], but evidence from the literature has subsequently raised doubts about their appropriateness. Analyses of small groups of healthy subjects initially suggested that platelet count varies by age [3] and sex [4], being higher in women than in men and in youth than in old age, with larger studies later confirming these findings [5], [9]. Additionally, significant differences in platelet count have been repeatedly observed in different ethnic populations [6], [10] and mainly attributed to genetic factors, which, however, remain largely unknown [8], [11], [12]. In particular, both the lower and the upper limits of the normal range seem to be inappropriate for the Italian population. Platelet count between 100 and 150×10^9^/L has been incidentally discovered in several Italian adults who subsequently did not develop any disorder during a 5-year follow up [13]. Moreover, the study of 12517 inhabitants of Sardinia geographic isolates revealed that in some villages the prevalence of mild thrombocytopenia was higher than 10% in old people, while in other villages the prevalence of mild thrombocytosis was 11% in subjects younger than 18 years [8].

Although data from the literature clearly indicate that the normal range presently in use is not appropriate, proposing reference intervals of platelet count that take into account gender and age, as well as ethnicity of people under evaluation, is a very ambitious goal that requires the investigation of tens of thousands of inhabitants. One such attempt was made in 2004 in the U.S. using data collected between 1988 and 1994 by the third National Health and Nutrition Examination Survey [14]. This study evaluated 20685 individuals from 64 sampling areas in the U.S., but excluded 12688 of them because of lifestyle characteristics or diseases, such as smoking, birth-control pill use, high body mass index, diabetes or hypertension, that could interfere with any of the various parameters under consideration. Mean platelet count and reference intervals were provided by sex, age (nine categories) and race (three categories). The trend of mean platelet count confirmed the variability due to gender, age and ethnicity, but the small number of subjects in each category resulted in erraticism of the lower and upper limits of normal ranges. As a matter of fact, these reference intervals for 54 different groups of people proved to be unsuitable for clinical practice and are not currently used.

Three population-based studies examining, among several other parameters, also platelet count have been recently published in Italy. Two evaluated 19783 residents in six geographic isolates located in different Italian regions [8], [9], and the third investigated 18097 inhabitants of Molise, a region in central Italy [7]. The availability of such a large amount of data from inhabitants of different geographical areas of one country prompted us to try not only to identify the normal ranges of platelet count for men and women and for different age groups, but also to measure the variability related to the different origins of the investigated populations. We present here the results of this study and propose new reference intervals for platelet count in Italy.

## Materials and Methods

### Ethics Statement

Study protocols were approved by the ethical committees of the different institutions who provided the data analyzed in this work (Ogliastra Genetic Park; Moli-sani Project; Cilento National Park; INGI-VB; South Tyrol Park; FVG genetic Park and Carlantino). All participants in the study gave their written informed consent for medical data collection, laboratory analysis, storage and use of biological material.

### Study sample and design

Our study protocol complied with the recommendations of the National Committee for Clinical Laboratory Standards (NCCLS) [15], [17] and the International Federation of Clinical Chemistry and Laboratory Medicine (IFCC) [18].

We analyzed data from seven studies carried out in Italy on different populations: 30 villages in Molise, in central Italy (n = 22969) [7], three villages of Cilento National Park, in south Italy (n = 2128) [19], [20], seven small villages of upper Borbera valley, localized within the Piedmont Apennines in the northwest of Italy (n = 1785) [21], the Carlantino village, situated in the south-eastern part of the Appennines in a hilly area of Puglia region (n = 1478) [22], five villages from the Genetic Park of Friuli Venezia Giulia (FVG), in north-eastern Italy (n = 1650) [23], three villages from South Tyrol, the northern-most Italian region (n = 1280) [24], and ten villages of the Ogliastra Genetic Park in Sardinia (n = 12517) [8]. Each study group supplied electronic records of their original data conforming to a standard protocol provided by the principal investigators. As in the original study, the Ogliastra villages have been merged into three groups (Northern, Western and Others) that were homogeneous in terms of genotype and phenotype characteristics of their inhabitants [8], [25], these are considered as separate entities, thus bringing to nine the total number of analyzed populations.

All these studies have similar characteristics in that they are large population-based epidemiologic surveys with a cross-sectional design carried out on the general population.

Detailed information on data collection, protocols and study design have been published elsewhere [7], [8], [19], [24]. Briefly, enrolled subjects (n = 43807, age range 0–105 years) provided socio-demographic and lifestyle data, as well as a detailed information on medical history and drug use, all collected through structured questionnaires. They underwent physical examination and blood sampling in local consulting rooms. Platelet count was determined by a Sysmex Hematology analyzer (DASIT, Cornaredo, MI, Italy) in the Borbera valley, and by Coulter Hematology analyzers (Beckman-Coulter, Brea, CA) in all other areas. Blood samples were collected in the morning and analysed within 3 hours after venipuncture. EDTA served as an anticoagulant.

In order to avoid biases due to the clinical phenotypes possibly affecting platelet count, subjects with overt liver disorders, neoplasia, or a family history of thrombocytopenia were excluded.

### Statistical analysis

In order to define optimal strata for reference interval estimation, platelet count was first explored graphically, then T-test and ANOVA on mean platelet count were used to assess differences by sex, age class and geographic area. Bonferroni correction was used to provide adjusted P-values and marginal means (*margins* command available in STATA 11) were computed to compare platelet count between strata taking into account the confounding effect of age and sex. Stratification aims at reducing variation thus narrowing intervals, though satisfying a minimum sample size of 120 statistical units within each strata, as recommended by Reed and coauthors [26]. Distribution of platelet count was explored to evaluate skewness, kurtosis, and to identify outliers. Reference intervals were estimated with the non parametric method computing the 2.5^th^ and 97.5^th^ percentiles. 95% confidence intervals were obtained with a binomial method that makes no assumptions about the underlying distribution of the variable [26]. Data management, quality control and, statistical analyses were performed by STATA 11 (College Station, TX).

## Results

Data from the different population-based studies on 43807 individuals were pooled together and checked for quality. After excluding subjects with overt liver disorders (3.9%), neoplasia (2.9%), or a family history of thrombocytopenia (0.12%), the sample size was reduced to 40987. Men were 46.2%, mean age was 50.7 years (SD 17.5) ranging from 10 months to 105 years. In South Tyrol and Borbera valley the minimum age was 18 years, and in Molise 35 years (Table 1).

**Table 1:** Characteristics of the pooled population-based studies' sample.

Population	n	Liver disorders (n)	Neoplasia (n)	Males (%)	Age range	Mean age (SD)	Mean PLT (SD)
**Carlantino**	1341	39	54	44	1–91	41.9 (21.6)	222 (56)
**North Ogliastra**	2151	95	56	43.9	3–98	44.1 (21.9)	232 (60)
**Borbera valley**	1641	89	63	44.2	18–102	54.3 (18.1)	241 (56)
**FVG**	1565	40	38	40.9	3–92	48 (20.1)	248 (68)
**Molise**	21599	692	715	48.3	35–99	55.5 (11.8)	250 (63)
**Cilento**	2059	22	50	44.7	0–103	48 (22.5)	251 (68)
**Others Ogliastra**	7081	420	150	44.6	2–105	43.8 (20.7)	254 (67)
**South Tyrol**	1168	81	34	43.5	18–94	45.5 (16.2)	267 (67)
**West Ogliastra**	2382	164	51	44.3	3–101	43.8 (22.2)	272 (66)
**Total**	40987	1642	1211	46.3	0–105	50.7 (17.5)	250 (65)

FVG, Friuli Venezia Giulia; PLT, platelet count.

**Figure 1:**
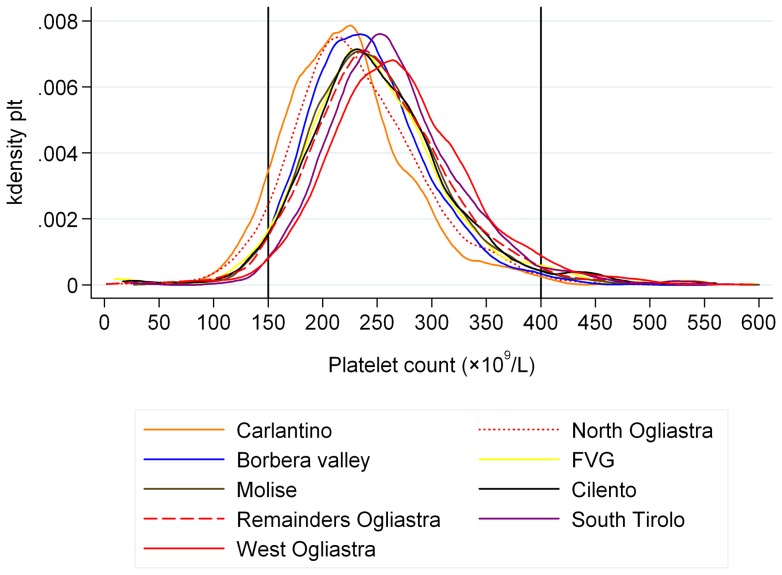
Platelet count densities by population. Each line represents the platelet count distribution of a population; Ogliastra villages have been clustered in three groups following their genotype and phenotype characteristics. Vertical lines represent the reference intervals currently in use.

Platelet count distribution was left-shifted in respect to the current reference interval and it was also remarkably different among populations, with the lowest mean value in Carlantino and the highest in West Ogliastra (Figure 1).

**Figure 2:**
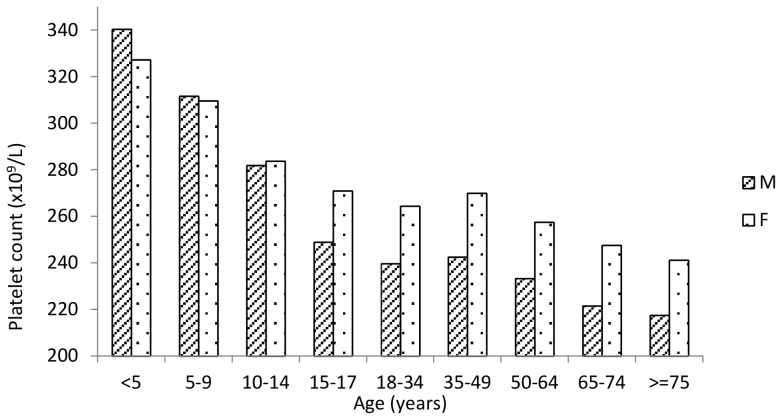
Platelet count by age in the examined populations.

**Figure 3:**
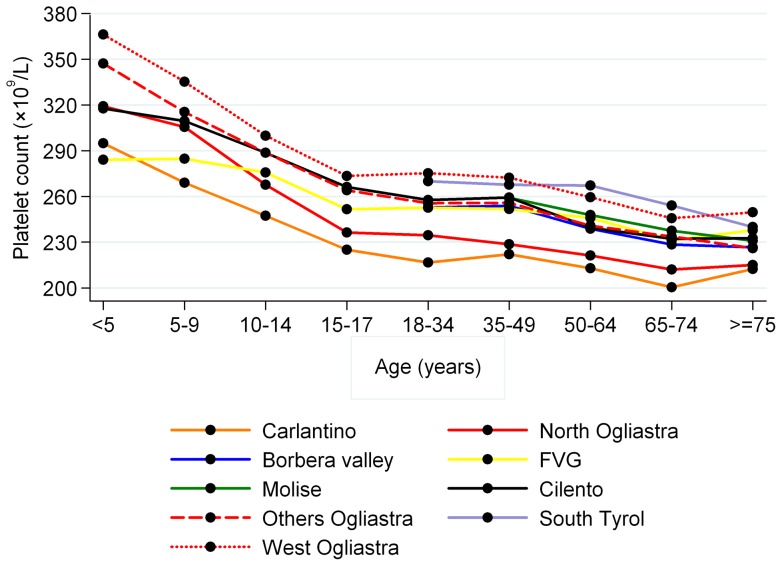
Platelet count by age and population. Lines represent age trend of platelet count in the investigated populations.

**Figure 4:**
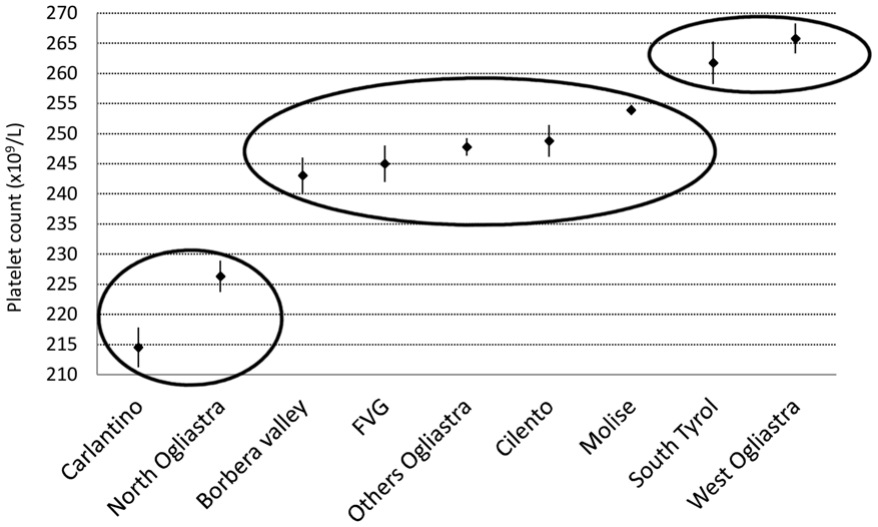
Mean platelet count in the nine sub-populations along with 95% CI. Estimates have been obtained by ANOVA adjusting for age and sex. Three strata may be obtained grouping sub-populations according to their mean platelet counts, as evidenced by black circles: Low (Carlantino and North Ogliastra), Medium (Borbera valley, FVG, Others Ogliastra, Cilento and, Molise) and, High (South Tyrol and West Ogliastra).

Overall, women had significantly more platelets than men (261 vs. 237×10^9^/L, *P*<0.001), and platelet count decreased with age, with a reduction from infancy to old age of 35% in men and about 25% in women (Figure 2). The observed age-related trend was common to all investigated populations (Figure 3). Under 15 years of age platelet count was significantly higher compared to the age range 15–64 years (299 vs 252×10^9^/L, *P*<0.001), and platelet count in the age range 15–64 years was significantly higher relative to subjects over 64 years (252 vs 233×10^9^/L, *P*<0.001). Of note, under 15 years of age there was no difference in platelet count of men and women (298 vs 299×10^9^/L, *P* = 0.690), whereas women had more platelets in the age range 15–64 years (264 vs 238×10^9^/L, *P*<0.001) and over 64 years (245 vs 220×10^9^/L, *P*<0.001). We therefore estimated platelet count reference intervals stratifying by three age classes and by sex for individuals over 14 years of age, as illustrated in Table 2. Mean platelet count varied significantly in different populations and, adjusting for age and sex, it ranged from 214 to 266×10^9^/L. Age- and sex-adjusted ANOVA, performed on the overall sample, showed significant differences among the average platelet count in different geographic areas (*P*<0.001). To combine this variability with the age- and gender-related differences in platelet count, distinct geographical areas have been grouped into three strata according to genotype and phenotype characteristics of their inhabitants: low-, medium- and high-platelet count areas, with mean platelet counts of 222, 251, and 265×10^9^/L, respectively) (Figure 4). The resulting age-, sex-, and population-specific mean platelet values, as well as the reference intervals, are reported in Table 3.

**Table 2:** Age- and sex-specific reference intervals with 95% CI for platelet count.

Age	n	mean (SD)	2.5^th^– 97.5^th^	2.5^th^ (95%CI)	97.5^th^ (95%CI)
**<15 years**					
All	1702	299 (71.6)	176–452	166–181	441–467
**15–64 years**					
Men	13789	238 (57.9)	141–362	140–144	358–365
Women	16358	264 (65.3)	156–405	153–158	401–410
**>64 years**					
Men	4303	220 (59.5)	122–350	119–126	343–360
Women	4835	245 (61.2)	140–379	137–144	372–390

SD, standard deviation; CI, confidence interval.

**Table 3:** Age-, sex- and, population-specific* reference intervals with 95% CI for platelet count.

	Low platelets areas					Medium platelets areas					High platelet areas				
Age	n	Mean (SD)	2.5^th^– 97.5^th^	2.5th (95%CI)	97.5th (95%CI)	n	Mean (SD)	2.5^th^– 97.5^th^	2.5th (95%CI)	97.5th (95%CI)	n	Mean (SD)	2.5^th^– 97.5^th^	2.5th (95%CI)	97.5th (95%CI)
**<15 years**															
All	426	277 (65.7)	165–412	144–176	396–441	995	301 (72.8)	179–459	162–185	441–484	281	322 (66.6)	196–473	181–218	452–503
**15–64 years**															
Men	1032	212 (52.6)	120–343	112–128	320–359	11614	239 (58)	143–362	141–146	358–366	1143	253 (54.9)	157–369	151–161	361–388
Women	1347	234 (54.2)	136–358	128–141	345–363	13577	265 (65)	157–405	156–160	401–410	1434	284 (68.6)	176–436	170–184	422–457
**>64 years**															
Men	279	201 (54.4)	112–332	73–123	304–363	3746	220 (59.5)	123–350	119–127	344–360	278	239 (58.8)	133–361	97–144	337–420
Women	408	217 (50.9)	119–325	103–133	307–349	4013	247 (61.4)	143–381	138–147	375–394	414	254 (60.5)	144–396	124–164	366–441

SD, standard deviation.

* Grouping of distinct geographical areas, according to their genotype and phenotype features, is as follows: Low (Carlantino and North Ogliastra), Medium (Borbera valley, FVG, Others Ogliastra, Cilento and, Molise) and, High platelets areas (South Tyrol and West Ogliastra).

## Discussion

Although several studies reported that platelet count decreases during aging, is higher in women than in men and varies in populations of different origin, this evidence has not been translated into the clinical practice due to the lack of appropriate reference intervals, and most laboratories in western countries still use the single reference interval 150 to 400 or 450×10^9^ platelets/L for all people.

To remedy this shortcoming for the Italian population, we took advantage from the availability of the databases of three recently published population studies including more than 40000 inhabitants of different Italian areas and examined closely age- and gender-related changes in platelet count to identify normal ranges of this parameter for different categories of subjects.

The large sample size of our study allowed us to investigate narrow age classes, and this approach revealed that age-related changes in platelet counts are very large: the number of platelets in old age is reduced by 35% in men and by 25% in women with respect to early infancy. Of note, most of this decrease occurs in childhood and, to a lesser extent, in old age, with only minor changes in adulthood (Figures 2, 3). The larger than expected age-related variability in platelet count further supports the need for age-specific reference intervals, as those reported in Table 2. Concerning the mechanisms that are responsible for the age-related changes, the sharp decrease of platelets during infancy may be related to the thrombopoietin levels that have been reported to decline from birth to adulthood [27], while the reduction in elderly people may reflect a reduction in hematopoietic stem cell reserve during aging or a survival advantage in subjects with lower platelet counts. However, these are only hypothesesand further investigation is required to identify the mechanisms underlying age-related changes.

The observation about platelet count decline in older age has been already reported in other studies, but for the first time now in a cohort as large as ours.

Another important finding of our study concerns gender-related differences in platelet count. Although we confirmed that, on average, women have significantly more platelets than men, we also showed that sex-related differences become relevant only after 14 years of age. From a practical point of view, this finding indicates that a single reference interval may be used for children, while separate intervals for men and women are required for older people. We believe that the age- and sex-specific reference intervals reported in Table 2 are a good compromise between accuracy and simplicity, and are therefore suited for use in clinical practice. Similarly to the age-related variability, the mechanisms responsible for sex-related differences in platelet count are also unknown. However, the observation that women begin to have platelet count higher than men only after the age of 14 supports the hypothesis that puberty makes the difference. Interestingly, a similar phenomenon, but in opposite direction, occurs for haemoglobin levels, which become lower in women than in men only after puberty. The reduction of body iron in menstruating women plays a role in their lower haemoglobin levels, but is probably related also to their higher platelet count in that moderate iron deficiency is known to stimulate platelets production [28], [29]. The gender-related difference in platelet count, as in haemoglobin levels, persists in the elderly and, once again, the level of iron may play a role, because also in old age it is lower in women [30]. However, the hormonal differences between men and women, which become larger after puberty, could also be involved. In particular, *in vitro* and *in vivo* findings showed that oestrogens favour platelets formation in mouse [31], but no data in humans are available.

Although we trust that the age- and gender-specific reference intervals reported in Table 2 represent a significant step forward for a proper interpretation of platelet count, they do not take into account the genetic and environmental factors that are known to influence the number of circulating platelets in the Italian population. A recent study in Sardinian geographic isolates well exemplifies the importance of these factors. The mean platelet count ranged from 214 to 266×10^9^/L in different villages, and the standardized prevalence of platelet count lower that 150×10^9^/L varied from 1.5% to 6.8% in different geographic areas, whereas the prevalence of platelet count higher than 400×10^9^/L ranged from 0.9% to 4.5% [8]. The heritability of platelet count, adjusted for age and sex, was 54% while environmental factors accounted for only 5% of phenotypic variance, suggesting that genetic factors had a major role in these differences. Still larger differences have been reported in another study that examined geographic isolates located in other Italian areas [9]. However, regional ranges for platelet count might only make sense for the few geographic isolates that still exist, since most modern populations in Western countries are characterized by high mobility and extensive admixture.

**Figure 5:**
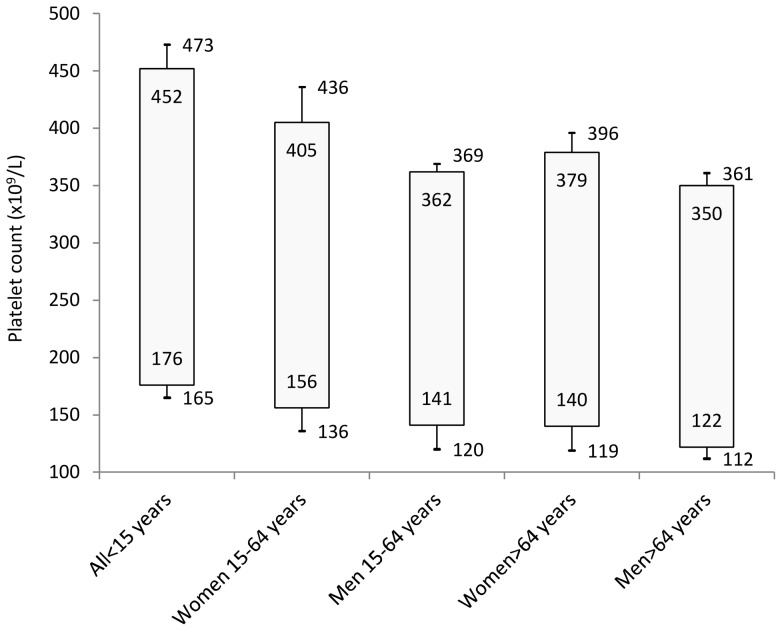
Platelet counts' reference intervals for clinical practice. Numbers inside bars represent reference intervals estimated on the overall sample; numbers outside bars represent extended reference intervals estimated stratifying by geographical area.

Genetic factors responsible for variation in platelet count in healthy individuals are being investigated and a few have been identified [11], [12], [32], but they account for only a little proportion of the phenotypic variance. Thus, definition of normal ranges taking into account people genotypes is not yet feasible. However, we can try to indirectly evaluate genotype-related variability by the differences in platelet count observed in different Italian geographic isolates. By this approach, we obtained the extended reference interval reported in Figure 5, whose upper and lower limits are those calculated in the isolates with the highest and the lowest mean platelet count, respectively. We propose that platelet count within the reference interval we estimated on the overall sample have to be considered normal, while those with platelet counts outside this reference interval but within the extended one are in an area of uncertainty that is still compatible with a normal phenotype, but is also compatible with the presence of illnesses that affect platelet count. Subjects in this grey area need a close follow up to decide between these two possibilities, since abnormalities of platelet count due to underlying, unrecognized diseases usually worsen over time, while the high or low platelet count of subjects with peculiar genetic backgrounds is expected to remain stable apart from the slow decline associated with aging.

This study has some potential limitations. A first weakness is the relatively limited number of the Italian regions whose inhabitants have been studied. It is therefore possible that inhabitants of other areas have higher or lower platelets count and that, as a consequence, the standard and the extended reference intervals we calculated are slightly different from the actual ones. In particular, it is possible that we under evaluated ethnicity-related platelet variability. Another limitation is that in South Tyrol, Borbera valley and Molise only subjects older than 17 and 34 years, respectively, have been examined. Thus, the lack of data of young people in these areas may have altered the evaluation of the influence of age on platelet count. This shortcoming could be particularly relevant for Molise, since inhabitants of this region represent more than half of the analysed population. However, the time course and mean values of platelet count in Molise inhabitants after the age of 34 years were similar to those observed in the whole population (data not shown), and thus we believe that this flaw has not had a significant effect on the assessment of age-related changes. Finally, the differences in platelet counts observed in different areas might have derived from different hematology analyzers used. However, only two different instruments were used for counting platelets in examined populations, both based on the impedentiometric technology. In particular, it has been previously reported that the two analyzers (Sysmex Hematology analyzer, used for Borbera valley inhabitants, and Coulter Hematology analyzer, used for all the other villages) gave similar results, especially in populations not including subjects with severe thrombocytopenia [33]. Moreover, although exactly the same instrument with identical operational settings was used for Sardinian inhabitants, much higher platelet counts were obtained in West Ogliastra with respect to North Ogliastra villages. These findings support the conclusion that the differences in platelet counts in different geographic areas were real.

In conclusion, analysis of pooled data of three population studies in Italy allowed us to identify age- and gender-specific reference intervals of platelet count, as well as variability of platelet count related to the different origins of investigated populations. The new reference intervals significantly differ from that currently used, especially concerning platelet count of children, who are presently at risk of receiving a wrong diagnosis of thrombocytosis, and old men, at risk of undue diagnosis of thrombocytopenia. We do not know if these ranges can be generalized to other populations. However, based on the few data available in the literature, it seems reasonable to hypothesize that they are not suitable for most areas of Africa [10], [34], [35], China [36], Iran [37] and Caribbean [10], where platelet counts seem to be lower than in Italy. Conversely, mean values of platelet count observed in Italy are similar to those observed in other Caucasian populations [6], and it is therefore possible that the reference intervals we propose for the Italian population are valid also in other Western countries.
